# Data‐driven MEG analysis to extract fMRI resting‐state networks

**DOI:** 10.1002/hbm.26644

**Published:** 2024-03-06

**Authors:** Esther A. Pelzer, Abhinav Sharma, Esther Florin

**Affiliations:** ^1^ Institute of Clinical Neuroscience and Medical Psychology, Medical Faculty Heinrich Heine University Düsseldorf Düsseldorf Germany; ^2^ Max‐Planck‐Institute for Metabolism Research Cologne Cologne Germany

**Keywords:** Envelope correlation, fMRI, ICA, MEG, phase‐amplitude coupling

## Abstract

The electrophysiological basis of resting‐state networks (RSN) is still under debate. In particular, no principled mechanism has been determined that is capable of explaining all RSN equally well. While magnetoencephalography (MEG) and electroencephalography are the methods of choice to determine the electrophysiological basis of RSN, no standard analysis pipeline of RSN yet exists. In this article, we compare the two main existing data‐driven analysis strategies for extracting RSNs from MEG data and introduce a third approach. The first approach uses phase–amplitude coupling to determine the RSN. The second approach extracts RSN through an independent component analysis of the Hilbert envelope in different frequency bands, while the third new approach uses a singular value decomposition instead. To evaluate these approaches, we compare the MEG‐RSN to the functional magnetic resonance imaging (fMRI)‐RSN from the same subjects. Overall, it was possible to extract RSN with MEG using all three techniques, which matched the group‐specific fMRI‐RSN. Interestingly the new approach based on SVD yielded significantly higher correspondence to five out of seven fMRI‐RSN than the two existing approaches. Importantly, with this approach, all networks—except for the visual network—had the highest correspondence to the fMRI networks within one frequency band. Thereby we provide further insights into the electrophysiological underpinnings of the fMRI‐RSNs. This knowledge will be important for the analysis of the electrophysiological connectome.

## Graphical abstract

We compare the two main existing data‐driven analysis strategies for extracting resting‐state network (RSN) from magnetoencephalography (MEG) data and introduce a third approach. While it is possible to extract RSN with MEG using all three techniques, the new approach yielded significantly higher correspondence for most RSN.

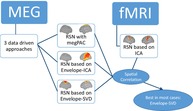

## 1 INTRODUCTION

During the past decade, a well‐reproducible connectivity map of brain activity during rest has been identified and thoroughly investigated in healthy humans using functional magnetic resonance imaging (fMRI) (Damoiseaux et al., 2006). Resting‐state analysis has gained increasing popularity in neuroscience because the data are relatively easy to acquire and do not depend on a task. Using magnetoencephalography (MEG), recent studies have started investigating the temporal dynamics of resting‐state networks (RSN) (Baker et al., 2014; Vidaurre et al., 2016). However, despite these advances, the electrophysiological underpinnings of the *canonical* fMRI‐RSN are still not completely understood as also discussed by Sadaghiani et al. (2022).

In this article, we aim to provide a rigorous comparison of different approaches to extract these canonical fMRI‐RSN from MEG data. We do so by comparing RSN extracted from resting‐state recordings for the same subjects in the MEG and fMRI. Using RSN from the same subjects allows us to eliminate potential biases stemming from variability in fMRI RSN. Another approach—not possible with MEG—is the simultaneous recording of electroencephalography (EEG) and fMRI in humans (Wirsich et al., 2021). In this study, the focus was, however, slightly different with the aim of comparing the overall connectivity matrix and not a comparison between extracted RSN.

Because the literature considers the networks extracted from fMRI as the gold standard, we take those as the benchmark and evaluate the MEG approaches according to their ability to match the fMRI results in the same subjects. In our comparison, we restrict our attention to data‐driven approaches and provide a further methodological advancement of the Envelope‐independent component analysis (ICA) approach (Brookes et al., 2011), which has been applied in numerous studies (Hall et al., 2013; Liu et al., 2017; Schneider et al., 2020; Wens et al., 2014). While seed‐based approaches can also be found in the literature (Hillebrand et al., 2012; Hipp et al., 2012; Marzetti et al., 2013), the seed choice adds a degree of freedom to the analysis that is difficult to control for. The ultimate goal of our study is to provide researchers with guidelines on how to extract canonical RSN in a data‐driven manner from MEG data.

The MEG‐RSN literature has provided non‐consistent findings on the main frequencies underlying individual RSN. For example, seed‐based envelope correlation ascribes the dominant frequency ranges for the default mode network (DMN) to theta and alpha, and for the dorsal attention network (DAN) the alpha and beta ranges (de Pasquale et al., 2010). In contrast, a data‐driven approach based on ICA of frequency‐specific envelopes (Envelope‐ICA approach, Brookes et al. (2011)) obtained the best correspondence for the DMN within the alpha band and for the DAN within the beta band. In this article, we investigate the role of RSN extraction techniques for potentially explaining some of these differences.

The phase lag index has also been proposed to obtain MEG‐RSN (Hillebrand et al., 2012; Marzetti et al., 2013; Stam et al., 2007) and this information might add further information to the seed‐based network estimation based on phase compared to amplitude alone (Tewarie et al., 2016). According to these studies, most of the functional connectivity in the tested RSN is promoted through alpha and beta oscillations. Unfortunately, these phase‐lag index studies have so far been limited to seed‐based analysis and no data‐driven approach exists for purely phase‐based resting‐state extractions. On the other hand, it was demonstrated with a data‐driven approach that phase‐amplitude coupling between a low‐frequency phase and high‐gamma amplitude can explain the formation of the fMRI‐RSN (Florin & Baillet, 2015) (megPAC approach)—thereby combining the information from amplitude and phase.

Within this article, we compare the different data‐driven approaches to extract RSN from MEG data based on resting‐state recordings from the same subjects in the MEG and fMRI. Their advantage is that they can be applied without any a priori assumptions on particular seed locations. Insights on the correspondence to fMRI‐RSN from such data‐driven approaches will be generalizable for future MEG studies and, therefore might provide important insights on the electrophysiological underpinnings of fMRI‐RSN.

## 2 MATERIALS AND METHODS

We included 26 healthy right‐handed male subjects. Three of these subjects had to be excluded due to movement artifacts or technical problems during data acquisition (23 subjects with age: 26.7 ± 3.9 SD; Edinburgh Handedness index 88.6 ± 20.7; mini mental status test: 29.8 ± 0.5). Before data acquisition, all subjects gave their informed consent and were then included in the experiment in line with the ethical guidelines of the declaration of Helsinki (Ethics committee Cologne: 14‐264, Ethics committee Düsseldorf: 5608R).

**FIGURE 1:**
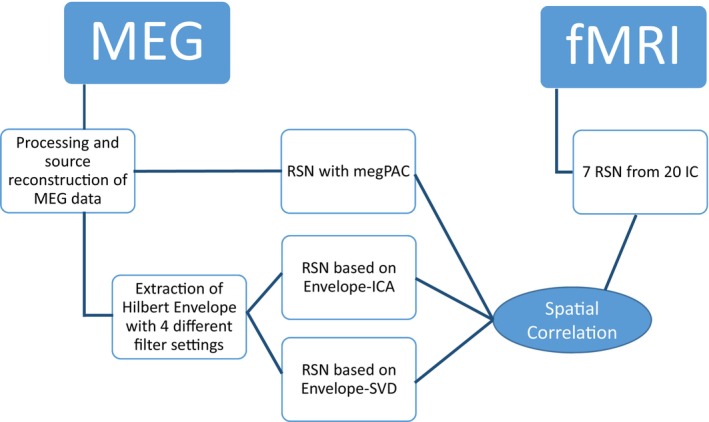
Flowchart of the analysis.

In the following, we will first explain the MEG data preprocessing and the RSN extraction, before describing the fMRI RSN extraction and our approach for comparing the two. The overall analysis outline is depicted in Figure 1.

### 2.1 MEG data acquisition and preprocessing

The MEG resting‐state data were acquired in a 306 channel MEG (Elekta‐Neuromag) system with a sampling rate of 2400 Hz and a 800 Hz anti‐aliasing filter. In total, 30 min of resting‐state activity in the MEG was recorded in a seated position for each subject. Subjects were asked to rest with eyes open and to look onto a fixation cross to reduce eye movement. The fixation cross was printed on paper and placed in front of the subject. This analog setup was used to exclude the possibility that the projector's refresh rate would lead to further extraneous frequency components (Logothetis et al., 2009). The MEG data were acquired in blocks of 10 min so that subjects could move in between the 10‐min blocks. Each 10‐min block should be long enough to capture the basic resting‐state fluctuations, as was recently recommended for MEG measurements (Liuzzi et al., 2017). To monitor the subject's head position, four head‐positioning coils were taped to the subject's scalp. The positions of the coils were measured relative to the subject's head using a 3D digitizer system (Polhemus Isotrack). For anatomical co‐registration with MRI, about 100 additional scalp points on the subject's scalp were also digitized. In addition to the MEG, we simultaneously recorded an electrocardiogram (ECG) and electrooculogram (EOG).

After data acquisition, the preprocessing of the MEG data was done with standard processes implemented in brainstorm (Tadel et al., 2011; https://neuroimage.usc.edu/brainstorm/, preprocessing and source reconstruction was done with brainstorm version of 10.08.2018). After the recording, the line noise and its harmonics were removed (50, 100, 150, 200, 250 Hz) with matched sinusoidals and sensors with high noise levels (based on their power‐spectrum) were excluded. The ECG and EOG were used to automatically detect eye‐blinks and heartbeats and to then remove them with signal space projectors. The data were then visually inspected for artifacts (muscle artifacts, head movements), with problematic time segments being excluded from further analysis. The cleaned MEG data were down‐sampled to 1000 Hz to reduce the amount of data.

A 5‐min empty‐room recording with the same sampling rate of 2400 Hz and an anti‐aliasing filter of 800 Hz, but with no subject present in the magnetically shielded room, was obtained on each recording day. The goal is capturing the sensor and environmental noise statistics. Based on these recordings the noise covariance matrices were calculated for use in the source estimation process.

Forward modeling of neural magnetic fields was performed using the overlapping‐sphere technique implemented in brainstorm (Huang et al., 1999). For the cortically constrained weighted minimum norm estimate (wMNE), the lead‐fields were computed from elementary current dipoles distributed perpendicularly to the individual cortical surface (Baillet et al., 2001). The individual surfaces were extracted with Freesurfer (version 5.3.0) using a tessellation of 15,000 (https://surfer.mnr.mgh.harvard.edu).

The MEG data preparation described in this section was common for the two compared approaches.

#### 2.1.1 Extracting the MEG RSNs

Once the source‐level data had been constructed, we extracted the MEG RSNs. We used the megPAC approach as described in Florin and Baillet (2015) and the Envelope‐ICA approach by Brookes et al. (2011). Both RSN extraction approaches first operate on the individual source‐reconstructed MEG‐data. To project the data from the individual to the standard anatomy for the cortical source model, Freesurfer's coregistered spheres were used as implemented in brainstorm (Fischl et al., 1999).

#### 2.1.2 megPAC approach

**FIGURE 2:**
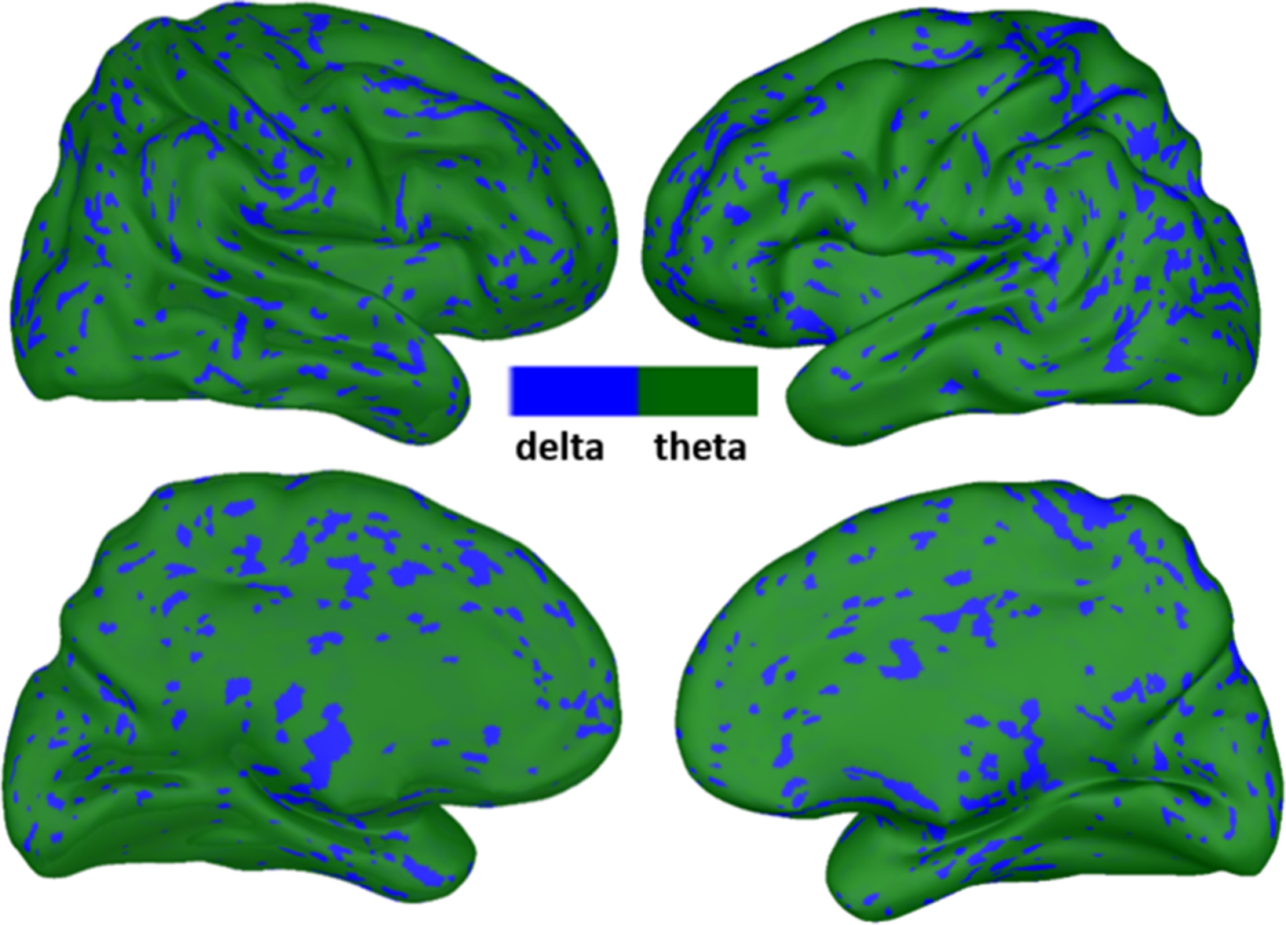
Low frequency of the megPAC approach. The cortical distribution of the low‐frequencies averaged across subjects is displayed. This is the frequency that exhibited the strongest coupling to the high gamma amplitude and was subsequently used to extract the resting‐state networks with the megPAC approach.

For the megPAC approach, we used the exact same parameters as described in the paper by Florin and Baillet (2015). First, for each source time series of each subject the low‐frequency phase that couples most strongly to the high gamma amplitude from 80 to 150 Hz was determined based on a phase‐amplitude coupling measure (Özkurt & Schnitzler, 2011). Figure 2 shows the average low‐frequency across subjects that exhibited the maximal phase‐amplitude coupling to the gamma amplitude in each subject. Similar to previous results, the low frequency was in the delta/theta range with no clear spatial pattern (Florin & Baillet, 2015). Both the phase and amplitude were extracted with a chirplet transform (Mann & Haykin, 1995), with a chirp factor of 0. For the low frequency corresponding to the identified phase of each individual vertex, the peaks and troughs were identified, and the gamma amplitude (80–150 Hz) was linearly interpolated between these events to 1000 Hz using the MATLAB function interp1. Through this process, for each source a new time series is obtained. The resulting time series were down‐sampled to 10 Hz and then projected to the Colin27 brain. Within the Montreal Neurological Institute (MNI) space the cortical time‐series from all subjects were first spatially smoothed (5 mm Gaussian Kernel) and then concatenated. Subsequently, the correlation matrix between all pair‐wise time‐series was calculated, yielding a connectivity matrix C of 15002 × 15002. Finally, the RSNs were determined as the principal spatial modes based on a singular value decomposition. To make this approach tractable each row of C was projected orthogonally on a subspace of 1175 cortically evenly distributed sources (Florin & Baillet, 2015). This approach was proposed in RSN fMRI by Yeo et al. (2011). This yields a new connectivity array P = C C^T^_1175_, where T stands for matrix transpose. To correct for the different sensitivity of the MEG recording depending on the cortical location, we also generated independent and identically distributed (i.i.d.) time series with zero mean and unit variance for each sensor with the same length as the original 26 data sets. Those time‐series were assigned a sampling frequency of 10 Hz and were accordingly low‐pass filtered by an anti‐aliasing filter. These data were then projected onto the Colin 27 brain using a precomputed Imaging‐Kernel for a wMNE of an Elekta system's sensor distribution. This yields a 15002*timepoints matrix, representing noise data on the cortical surface. Those were then spatially smoothed (5 mm Gaussian Kernel) and the correlation matrix between all pair‐wise time‐series was calculated. This correlation matrix was also projected onto the subspace of 1175 cortically evenly distributed sources. From this reduced connectivity array the first singular mode from a singular value decomposition was used to create an orthogonal projector $\tilde{\pi }$. The connectivity array P was multiplied by this projector, that is, $P\tilde{\pi }$ and with a singular value decomposition the RSNs were identified as U: $P\tilde{\pi }={\text{USV}}^{T}$ (see Florin & Baillet, 2015). These RSNs were compared to the fMRI RSNs obtained from the same subjects.

#### 2.1.3 Envelope‐ICA approach

For the Envelope‐ICA approach by Brookes et al. (2011), the Hilbert envelope is calculated in the five most common electrophysiological frequency bands: delta (1–4 Hz), theta (4‐8 Hz), alpha (8–12 Hz), beta (12–30 Hz), and gamma (30–50 Hz). We restricted our analysis to the approach by Brookes et al. (2011), except for also testing the effect of variations in filter settings and the ICA approach. To extract the envelope data for these five frequency bands we tested two different variants of filtering the data to obtain the Hilbert envelope:

1. Wide band: Filter width based on the predefined frequency bands (delta, theta, alpha, beta, and gamma). After filtering, the data were Hilbert transformed to obtain the envelope. This is the original approach described in Brookes et al. (2011).
2. Small band: From 1 to 4 Hz, we band‐pass filtered the data in 1 Hz bins, from 4 to 30 Hz in 2 Hz bins, and above 30 Hz in 5 Hz bins. The reason for this approach is that, based on the typical 1/f characteristics in wide frequency bands, the lower frequencies will dominate the envelope estimation. The resulting filtered time‐series were Hilbert transformed to obtain the envelope. To obtain a single time series for the whole frequency band, we standardized the envelope data by z‐scoring for each frequency bin and then averaged across the frequency bins within the respective band.

For each of the two binning approaches to obtain the Hilbert envelope we tested two different filter types: an infinite impulse response (IIR) filter and a finite impulse response (FIR) filter, both as implemented in the brainstorm function bst_bandpass_filtfilt. Both the IIR and the FIR filter were tested with the wide band and small band, resulting in four different filter combinations: small band FIR, small band IIR, wide band FIR, and wide band IIR filter.

Once the Hilbert envelope data at the individual level were obtained for each of the four filtering combinations, they were down‐sampled to 1 Hz and projected onto the Colin27 brain. On the template brain the individual cortically constrained data were spatially smoothed with a 5 mm Gaussian Kernel and then z‐scored in the time‐domain. The data from all subjects were subsequently concatenated in each frequency band. To extract the RSN, we follow Brookes et al. (2011) and employ the fastICA on the pre‐whitened data based on a dimensionality reduction to 30 principal components. Because the reliability and numerical stability of one single ICA‐run is usually not known, we performed an additional analysis using the ICASSO algorithm with random initialization to extract the temporal independent components of each frequency band (Himberg et al., 2004). For the ICASSO calculation, we used the toolbox GroupICATv4.0b. In the ICASSO algorithm, the fastICA was run 500 times on the pre‐whitened data. The selection of the ICASSO parameters is based on the suggestions from (Nugent et al., 2015). For the pre‐whitening, a principal component analysis was used to reduce the dimensionality to 30 components. The resulting independent components from each ICASSO run were then clustered based on the absolute value of the linear correlation coefficient between components (Himberg et al., 2004). For these clusters the centrotype, which is the estimate that best represents all other estimates in the same cluster, was estimated and used for further calculations. To obtain spatial RSNs, the temporal independent components (the centrotype in case of ICASSO) were correlated with the envelope data. These correlation maps for each of the analyzed frequency bands were compared to the resting‐state maps based on the fMRI data.

#### 2.1.4 Envelope‐SVD approach

In addition to extracting the RSNs from the envelope data with ICA, we also tested an SVD similar to the megPAC approach. This Envelope‐SVD approach uses the same approach for the extraction of the Hilbert envelope as the Envelope‐ICA approach, including the down sampling to 1 Hz and the projection to the standard brain. To extract the RSN from the Hilbert envelope data, however, the approach of the megPAC method is used: the temporal correlation between all envelopes within a given frequency band was calculated, yielding a 15,002 by 15,002 correlation matrix. The dimensionality of this matrix was reduced to 1175 patches in the same manner as for the megPAC approach. The RSNs within each frequency band were extracted as the principal modes of these correlation matrices based on a SVD. We also performed a noise reduction based on i.i.d. sensor data as described for the megPAC approach above and in Florin and Baillet (2015). This noise reduction was done for each frequency band separately.

### 2.2 MRI data acquisition and extraction of RSNs

All magnetic resonance imaging data were obtained using a Siemens 3 T PRISMA scanner using a 64‐channel head coil. The high‐resolution T1‐weighted images were acquired by applying a 3D MPRAGE sequence (TR = 2300 ms, TE = 2.32 ms, ES = 7.2 ms, FA = 8°, FOV = 230 mm × 230 mm, isotropic pixel resolution of 0.9 × 0.9 × 0.9 mm, slice thickness of 0.9 mm, 192 slices). Resting fMRI data were recorded with echo‐planar‐imaging acquisition, (TR = 776 ms, multiband acceleration of 8, TE = 37.4 ms, flip angle = 55°, resolution 2.0 × 2.0 × 2.0 mm, slice thickness of 2.0 mm, 72 slices). The resting fMRI scan lasted 30 min. Subjects were also asked to rest with eyes open and to fixate on a paper cross to reduce eye movement.

T1‐weighted images were automatically preprocessed with Freesurfer version 5.3.0 (recon‐all) in order to extract the brain and cortex surface; brain extraction performed with Freesurfer yielded better results in the differentiation of cortical areas from the skull than FSL‐BET. The resulting skull‐stripped T1‐weighted datasets were used as reference images in MELODIC 3.0 (https://fsl.fmrib.ox.ac.uk/fsl/fslwiki/MELODIC) after affine registration to standard MNI 2 mm space via FLIRT, a registration tool in FSL 5.0 (Jenkinson & Smith, 2001).

Data preprocessing of the fMRI 4D images was further carried out with FSL tools and the results were visually inspected. The following preprocessing was applied for each subject using MELODIC's Pre‐Stats feature: head motion correction via MCFLIRT (Jenkinson & Smith, 2001); removal of non‐brain areas using BET (Smith, 2002), spatial smoothing with a Gaussian kernel of full width at half maximum 4 mm; grand‐mean intensity normalization of the entire 4D dataset by a single multiplicative factor; 100 s high‐pass temporal filtering.

Registration of each subject's fMRI data to that subject's high‐resolution structural image was carried out by using 6 degrees of freedom registration with FLIRT (Jenkinson & Smith, 2001). Registration to the high‐resolution structural MNI‐152‐2‐mm standard space was achieved by using FLIRT affine registration.

For the cortically constrained analysis, we used the binarized cortical mask obtained from Freesurfer for the MEG case to restrict the fMRI ICA analysis to those cortical areas. We chose to variance‐normalize time courses to make sure that mere differences in the voxel‐wise standard deviations do not bias the PCA step and ICA cost function. Consistent with our MEG analysis, 20 ICA components were computed on data temporally concatenated across subjects.

After ICA decomposition, we chose the standard threshold of 0.5 for the IC maps following the recommendations in FSL Melodic. A threshold level of 0.5 in the case of alternative hypothesis testing means that a voxel “survives” thresholding as soon as the probability of being in the “active” class (as modeled by the Gamma densities) exceeds the probability of being in the “background” noise class (see https://fsl.fmrib.ox.ac.uk/fsl/fslwiki/MELODIC).

In order to compare the fMRI RSNs with MEG RSNs, we finally registered IC components, located in the MNI‐152‐2mm standard space, to the Colin27 brain.

### 2.3 Comparison of the fMRI and MEG RSNs

In accordance with the previous literature, we consider the fMRI RSNs to be the benchmark and judge the success of the MEG RSN identification approaches based on their ability to reproduce these fMRI networks.

To make the range of values across networks and approaches (in particular to the fMRI scaling) comparable, we assign to each of the 15,002 vertices in the MEG networks the cumulative probability of its value, that is, the likelihood of the respective vertex value within a given network vector being at least as big as its current value. In the case of megPAC and Envelope‐SVD, there is a natural mass point at 0 that is excluded before computing the CDF.

To identify the best‐matching MEG maps for both the megPAC and Envelope‐ICA approach as compared to the fMRI maps relies on the spatial correlation between the MEG and fMRI RSN. As the fMRI maps are provided in volumetric nifti format, we also converted our vertex‐based MEG results into volumetric nifti files with the same resolution as the fMRI networks using dedicated conversion functions within brainstorm. The correlation was calculated across all voxels, which matched the cortical mask, between the RSN from the two modalities. For each fMRI RSN we selected the best matching MEG component with the highest spatial correlation. In this case, neither the fMRI nor the MEG maps were thresholded.

### 2.4 Statistics for the comparison of the MEG approaches

To statistically compare the different approaches to extract the RSNs, we bootstrapped the envelope time series of each frequency 100 times across subjects with replacement for the Envelope‐ICA and Envelope‐SVD approach and the megPAC time series 100 times for the megPAC approach. This yielded us new envelope/megPAC time‐series of 23 randomly selected subjects. Afterward, the networks were extracted for each method as described above and the spatial correlation was computed for each of those repetitions. This provides a statistical distribution that captures sampling variability and allows for statistical inference. We first used a two‐way ANOVA to determine whether the bandwidth of the filter or the chosen filter type have a significant influence on the network estimation when employing the Envelope‐ICA and Envelope‐SVD. We used Bonferroni correction to correct for the seven networks tested and report only those results as significant which are lower than *p* = .05 after Bonferroni correction. Using post hoc tests with Bonferroni correction, we then identified significant differences based on the filter choice. To compare the Envelope‐ICA, Envelope‐SVD, and the megPAC method, we used the best filter setting for the Envelope‐ICA and Envelope‐SVD and performed a one‐way ANOVA with a two‐sided post hoc *t* test using Bonferroni correction to determine the method with the significantly highest spatial correlation for each network.

## 3 RESULTS

**FIGURE 3:**
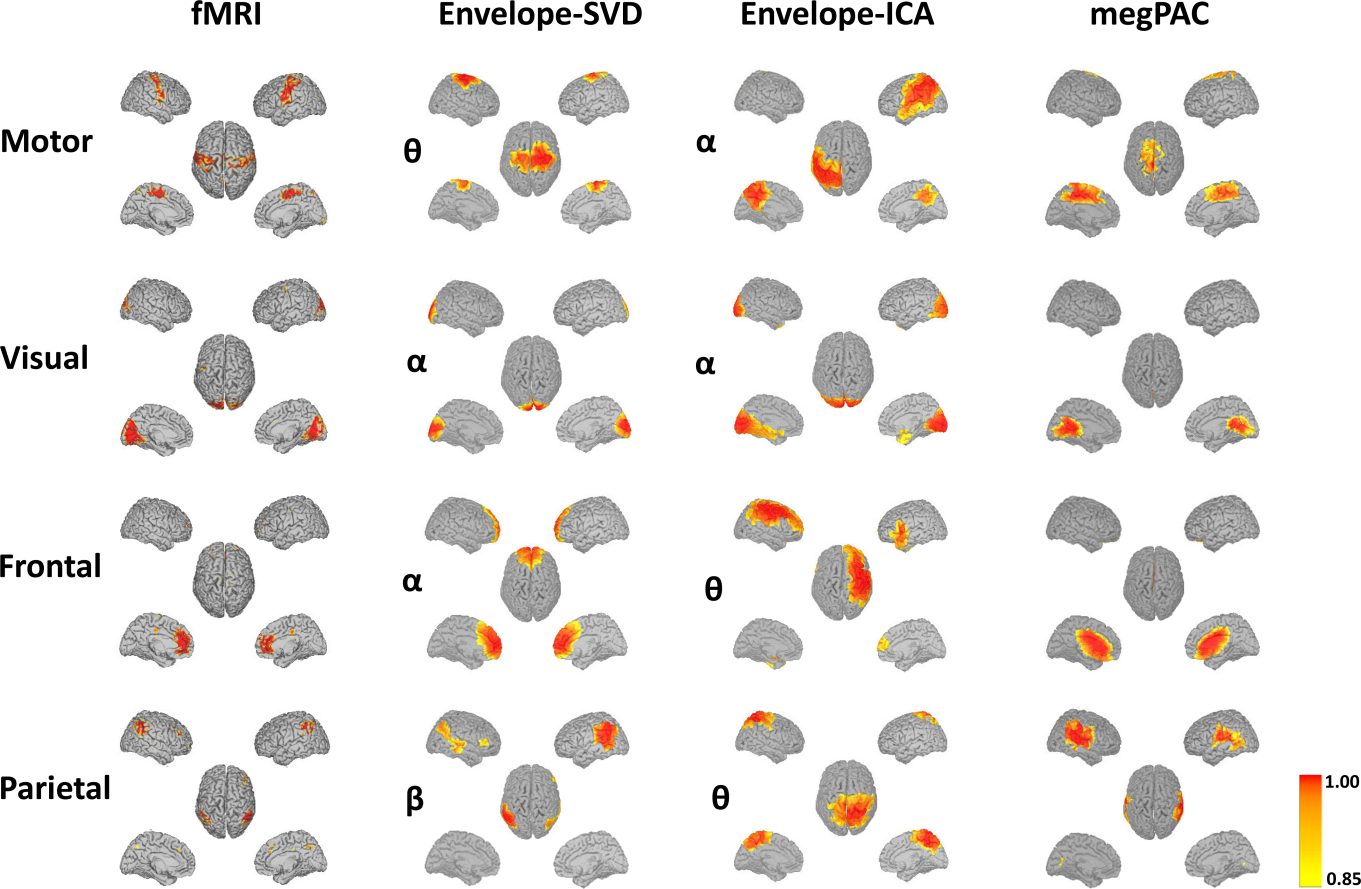
Highest correspondence resting‐state networks obtained from magnetoencephalography (MEG) and functional magnetic resonance imaging (fMRI) recordings using the three different approaches. For each MEG method, the resting‐state network (RSN) with the largest spatial correspondence to the fMRI‐RSN across all frequency bands and extracted networks is displayed. The row represents the respective RSN, while the columns show the spatial extent comparing fMRI to each of the three MEG approaches. The frequencies for the Envelope‐SVD and Envelope‐independent component analysis (ICA) approach are written on the left of each network based on the average best frequency across the 100 bootstrap repetitions as displayed in Figure 4. Note also that the results are based on ICA in this figure. The networks are all thresholded at 0.85 based on the probability distribution of the network's values.

In total, we extracted 20 ICs from the fMRI and MEG data. From the fMRI data, we used seven RSNs for the further comparison: the frontal, parietal, left and right front‐parietal, motor, visual, and DMN (see Figure 3). While motor, visual, DMN, and frontoparietal network match with the canonical definition of RSN (Yeo et al., 2011), the frontal and parietal network were named based on their anatomical locations, as there was no clear correspondence to those in Yeo et al. (2011). Of note, the frontoparietal network was separated into a left and right component in our analysis—likely because we calculated 20 ICs. In addition, the visual network in our case did not contain V1, but was mainly located in V2 and V3. Such variations can happen due the sample selection as well as the chosen number of ICs. This is also the reason why we chose to extract the fMRI and MEG RSN from the same participant pool to account for such variability. In the following, we will quantify in more detail the correspondence between the MEG‐RSN and fMRI‐RSN as well as several crucial choices for the Envelope based approaches.

### 3.1 Choice of filter for the envelope‐based approaches

When extracting the Hilbert envelope for the Envelope‐ICA and Envelope‐SVD approach, different choices for the filter settings can be made. As described in the methods section, four different filter settings were tested. Using an ANOVA, we identified that the filter type had a significant influence with a medium effect size on the spatial correlation for all networks in the case of Envelope‐SVD. This was accompanied by a significant interaction with the chosen frequency band. In the case of Envelope‐ICA, the filter type had only in three cases a significant but weak influence (see Table 1 for the corresponding *F*‐values and effect sizes).

**TABLE 1:** Effect of filter type on network identification.

	SVD									
	Filter			Frequency			Filter × frequency			Best filter
	*F*	*η* ^2^	*ƒ*	*F*	*η* ^2^		*F*	*η* ^2^	*ƒ*	
Visual	106.45	0.05	0.22	709.75	0.42	0.85	135.84	0.24	0.56	Wide IIR
Frontoparietal‐l	118.90	0.09	0.32	140.69	0.15	0.42	72.76	0.23	0.55	Small FIR
Frontal	246.65	0.06	0.26	1756.80	0.59	1.21	175.94	0.18	0.47	Wide IIR
Motor	449.76	0.25	0.58	170.58	0.13	0.38	114.41	0.26	0.59	Wide FIR
DMN	106.73	0.09	0.32	223.55	0.26	0.60	17.75	0.06	0.26	Wide FIR
Parietal	97.76	0.03	0.19	1393.93	0.66	1.40	48.56	0.07	0.27	Wide IIR
Frontoparietal‐r	82.18	0.08	0.30	28.20	0.04	0.20	55.85	0.22	0.54	Wide IIR

	ICA									
	Filter			Frequency			Filter × frequency			Best filter
	*F*	*η* ^2^	*ƒ*	*F*	*η* ^2^	*ƒ*	*F*	*η* ^2^	*ƒ*	
Visual	10.21	0.01	0.12	55.01	0.10	0.33	3.50	0.02	0.14	Wide IIR
Frontoparietal‐l	0.26 (n.s.)	0.00	0.02	5.37	0.01	0.10	1.53 (n.s.)	0.01	0.10	Small FIR
Frontal	2.95 (n.s.)	0.00	0.07	7.03	0.01	0.12	3.12	0.02	0.14	Small IIR
Motor	19.84	0.03	0.17	30.22	0.05	0.24	5.62	0.03	0.18	Wide FIR
DMN	3.38 (n.s.)	0.00	0.07	32.88	0.06	0.25	4.82	0.03	0.17	Wide IIR
Parietal	4.15	0.01	0.08	23.08	0.04	0.22	1.76 (n.s.)	0.01	0.10	Wide FIR
Frontoparietal‐r	3.29 (n.s.)	0.00	0.07	5.63	0.01	0.11	2.31	0.01	0.12	Wide IIR

*Note*: For each network, the main effect (*F*‐value) based on an ANOVA for the filter choice, frequency, and the interaction are reported. The nonsignificant effects after Bonferroni correction are marked with n.s. Moreover, we report the effect size *η* ^2^ and *ƒ*. *η* ^2^ was computed with the effect size toolbox in MATLAB (Hentschke & Stuttgen, 2011) and based on this *ƒ* was computed as provided in G*Power (Faul et al., 2007). In addition, the filter choice is reported, which led, in combination with the frequency band, to the highest correspondence with the fMRI network.

Next, post hoc analysis was performed to identify the filter combination, which yielded the highest correspondence to the fMRI network. Of note, this is only an absolute evaluation of the correlation value and was not based on significance testing, that is, the highest corresponding filter setting is not necessarily the significantly highest. This analysis revealed that the wide‐band filter yielded higher correspondence to the fMRI resting‐state maps for six networks in the case of SVD and for five networks in the case of ICA. Concerning the use of an IIR or FIR filter, the IIR filter led to significantly better correspondence to the fMRI networks for four out of seven networks. Based on this, all following results will be presented for a wide‐band IIR filter—even though the choice of IIR and FIR filter does not have a large effect.

### 3.2 Comparison of the different RSN extraction techniques

**FIGURE 4:**
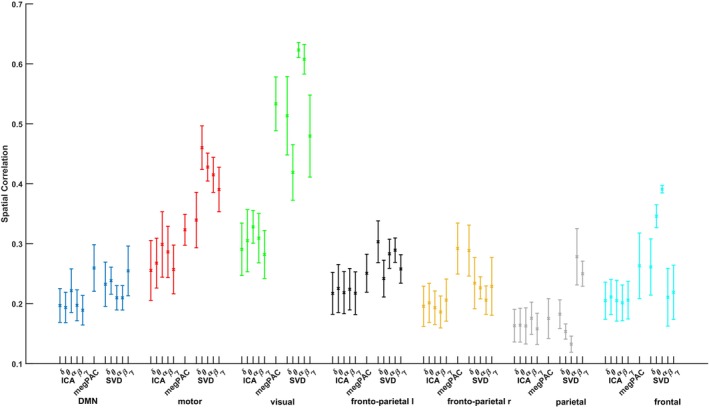
Spatial correlation between functional magnetic resonance imaging (fMRI) and magnetoencephalography (MEG) resting‐state network (RSN). The Envelope‐independent component analysis (ICA)‐based results were obtained with the optimal filter setting of a wide band IIR filter (see methods for details). The different frequency bands for the Envelope‐ICA approach, megPAC, and the Envelope‐SVD approach are plotted on the x‐axis and are grouped based on the seven tested resting‐state networks. The y‐axis depicts the spatial correlation −1≤ρ≤1 between the fMRI‐RSN and the corresponding MEG‐RSN for the respective method. The whisker plots indicate the mean and 1 SD across bootstrap repetitions.

As an example for the actual RSNs, we show four networks along the rows of Figure 3—all networks including each network within each frequency band for the Envelope‐ICA and Envelope‐SVD approach are shown in Supplementary Figures 1–3. The left column in these figures depicts the fMRI network, the right columns the matched MEG network for each method according to spatial correlation. Please note that for the Envelope‐ICA and Envelope‐SVD approach the displayed network is for the frequency‐band which on average yielded the best correspondence to the fMRI network, as seen in Figure 4. This also implies that, for example, in the case of the Envelope‐ICA approach the motor network is displayed in the alpha‐band, while as can be seen in Supplementary Figure 3 for this particular ICA run the best spatial correspondence to the fMRI network was obtained in the gamma frequency range. Overall, three qualitative things stand out. First, the MEG networks from all three approaches resemble the fMRI networks. Second, the spatial extent of the networks is largest for the Envelope‐ICA approach. Third, megPAC and Envelope‐SVD result in networks of similar spatial extent, but the latter better matches those of the fMRI data. This is particularly true for the motor, visual, and parietal network.

To more formally quantify the correspondence between fMRI and MEG networks across approaches, we calculated their spatial correlation (Figure 4). Therefore, we first evaluated the performance of all three approaches. To have a quantitative criterion of performance we calculated the average correlation across all seven networks for each method. For each network, we used the frequency with the highest average spatial correlation to the fMRI networks. Based on a one‐way ANOVA and post hoc testing, the Envelope‐SVD yields a significantly higher spatial correlation than both other methods and the megPAC has a significantly higher spatial correlation than then Envelope‐ICA approach (spatial correlation [mean ± std]: Envelope‐SVD: 0.37 ± 0.13, Envelope‐ICA: 0.24 ± 0.06, megPAC: 0.30 ± 0.11; one‐way ANOVA: *F* = 284.11, dof = 2, *p* = 6.9091e‐110). In addition, for all networks, there was a significant main effect of the chosen methods (see Table 2).

**TABLE 2:** Influence of chosen method and frequency on networks for wide IIR filter.

	All methods and frequencies			Frequency ICA			Frequency SVD		
	*F*	*η* ^2^	*ƒ*	*F*	*η* ^2^	*ƒ*	*F*	*η* ^2^	*ƒ*
Visual	829.26	0.88	2.76	18.06	0.13	0.38	314.36	0.72	1.59
Frontoparietal‐l	97.33	0.47	0.95	1.15 (n.s.)	0.01	0.10	82.74	0.40	0.82
Frontal	308.87	0.74	1.69	1.26 (n.s.)	0.01	0.10	455.51	0.79	1.92
Motor	388.44	0.76	1.77	16.60	0.12	0.37	163.51	0.57	1.15
DMN	67.12	0.38	0.79	19.78	0.14	0.40	42.21	0.25	0.58
Parietal	243.34	0.69	1.50	5.52	0.04	0.21	548.92	0.82	2.11
Frontoparietal‐r	107.27	0.50	0.99	5.79	0.05	0.22	69.49	0.36	0.75

*Note*: Using the previously identified filter option (wide IIR) we provide for each network the main effect (*F*‐value) based on a one‐way ANOVA for all methods and frequencies. The nonsignificant effect after Bonferroni correction is marked with n.s. Moreover, we report the effect size *η* ^2^ and *ƒ*.

Summarizing our findings, the Envelope‐SVD approach yielded the significantly best correspondence for all networks except for the DMN and the right frontoparietal network, for which the megPAC had the highest correspondence. However, this difference is not significant compared to the Envelope‐SVD (post hoc two‐sided *t* test *p* > .05, Bonferroni corrected). In regard to the frequency band, there is a significant effect on the correspondence with the fMRI maps for both the Envelope‐ICA and the Envelope‐SVD approach (see Table 2 for the *F*‐values). However, in case of the Envelope‐ICA, for the frontal and frontoparietal left network, there was no effect of the frequency. Moreover, a single dominant frequency band where the spatial correlation was significantly higher than in all other bands could only be identified for the DMN and visual network with alpha and the parietal network with beta (post hoc two‐sided *t* test *p* < .05, Bonferroni corrected). In contrast, for the Envelope‐SVD there was a clear frequency specificity, that is, one frequency had significantly higher correspondence than all other frequencies, for all networks except for the visual network (Figure 4), where both the alpha and beta range had a significantly higher correspondence than the other networks.

To further identify the frequency contribution to each RSN, we also performed a linear regression model as described and summarized in Table 3. The estimated coefficients for the different frequency components align with the spatial correlation results, that is, the frequency with the highest average correlation has also the highest regression coefficient. As expected the explanatory power of the frequencies overlap and thus the coefficients for the other frequencies are smaller than the reported spatial correlation. Also the *R* ^2^ of the linear regression reflects the visual impression on which networks have a better or lesser correspondence with the fMRI RSNs.

$${\mathrm{RSN}}_{\text{fMRI}}=a*{\mathrm{RSN}}_{\mathrm{MEG}}^{\delta }+b*{\mathrm{RSN}}_{\mathrm{MEG}}^{\theta }+c*{\mathrm{RSN}}_{\mathrm{MEG}}^{\alpha }+d*{\mathrm{RSN}}_{\mathrm{MEG}}^{\beta }\;+e*{\mathrm{RSN}}_{\mathrm{MEG}}^{\gamma }+\text{noise}.$$

**TABLE 3:** Linear regression model. Below the regression coefficients as well as the *R* ^2^ are reported.

	*a*	*b*	*c*	*d*	*e*	*R* ^2^
*SVD*						
Visual	0.005	0.009	0.759	0.130	0.007	.388
Frontoparietal‐l	0.178	0.068	0.105	0.127	0.110	.115
Frontal	−0.015	0.076	0.299	0.066	0.052	.161
Motor	0.045	0.337	0.172	0.149	−0.015	.229
DMN	0.065	0.093	0.019	0.085	0.179	.080
Parietal	0.058	0.028	−0.022	0.190	0.076	.086
Frontoparietal‐r	0.211	0.021	0.096	0.025	0.071	.099
*ICA*						
Visual	0.090	0.116	0.168	0.104	0.065	.142
Frontoparietal‐l	0.090	0.102	0.082	0.105	0.081	.075
Frontal	0.068	0.073	0.065	0.056	0.068	.068
Motor	0.062	0.090	0.158	0.128	0.063	.115
DMN	0.070	0.062	0.122	0.071	0.063	.067
Parietal	0.051	0.042	0.048	0.067	0.045	.045
Frontoparietal‐r	0.070	0.079	0.055	0.049	0.090	.064

*Note*: In addition to the spatial regression shown in Figure 4, we also fit a linear regression for each RSN to determine the frequency contribution to each RSN.

### 3.3 ICASSO for Envelope‐ICA

**FIGURE 5:**
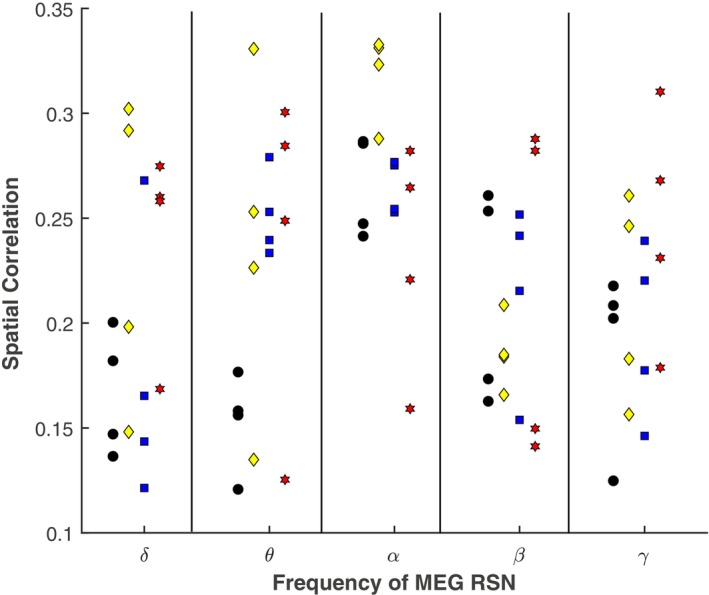
Independent component analysis (ICA)‐induced variability of spatial correlation for the Envelope‐ICA approach for four resting‐state networks (color coded). Within each frequency band, four repetitions of the ICASSO algorithm were conducted. The spatial correlation between the functional magnetic resonance imaging (fMRI) and magnetoencephalography (MEG) map is displayed. Note the variability across repetitions (black circle: default mode network [DMN], yellow diamond: motor, blue square: frontoparietal left, red star: visual). The different frequency bands for the Envelope‐ICA approach are plotted on the x‐axis. On the y‐axis, the spatial correlation between the fMRI resting‐state network and the corresponding MEG resting‐state network is plotted.

The Envelope‐ICA requires identifying independent components for each frequency band. However, as ICA is a high‐dimensional optimization problem, the reliability of a single ICA run is not known (Eriksson & Koivunen, 2004; Hyvarinen & Sabo, 2013), because this is dependent on the initial seed. Therefore, to stabilize the results, we ran the ICASSO algorithm four times for each frequency band. We then investigated whether the frequency components characterizing the individual networks are consistently the same across repetitions. When determining the IC map best matching the fMRI map, the spatial correlation value varied, resulting in ICs from different frequency bands showing the best (qualitative) correspondence to a particular fMRI RSN. An example is provided in Figure 5 for four RSNs when using the wide‐band IIR filter settings. Within this figure, the spatial correlation between the fMRI RSNs and these four RSNs obtained with Envelope‐ICA for the five frequency bands is plotted. For each RSN, the spatial correlation values for the four ICASSO repetitions are provided. As can be seen from the figure, these are highly variable across repetitions. Given these variable results, assigning the best match to a particular frequency band seems arbitrary. Importantly in this section, no statistical test is performed between the four repetitions. This is, however, the same as when one would run an ICA analysis and then select a network within one frequency band which qualitatively best matches an fMRI RSN.

## 4 DISCUSSION

Within this article, we propose a novel data‐driven approach to extract RSNs from MEG and compare it with other data‐driven approaches to extract RSNs from MEG data as similar as possible to fMRI. To minimize distortions introduced by RSN variability at the group level, the comparison was made on recordings of the same subjects once in the MEG and once in the fMRI. This is in contrast to previous studies employing standard fMRI maps for their comparison (Brookes et al., 2011; Florin & Baillet, 2015). The performance of MEG‐RSN approaches was evaluated based on the spatial correspondence to the fMRI‐RSN maps. Concerning the use of a method the correspondence between fMRI‐RSN maps and MEG‐RSN maps was significantly highest with the novel Envelope‐SVD approach followed by the megPAC approach, which yielded similar results for two networks. Furthermore, when employing the Envelope‐ICA or Envelope‐SVD approach, the filter settings affected the results: A wide‐band IIR band‐pass filter provided the best results. Concerning the physiological underpinnings of the fMRI‐RSN, we demonstrate that anatomically confined RSNs are frequency‐specific, while distributed RSNs seem to be communicating within multiple frequencies.

### 4.1 Choice of method

The Envelope‐SVD approach provided the highest correspondence to the anatomically confined fMRI networks. Moreover, the Envelope‐SVD shows a better fit to the fMRI networks than the Envelope‐ICA, has a unique solution, and exhibits a clear frequency specificity.

An interesting exception to the superior performance of the Envelope‐SVD approach are the DMN and the right frontoparietal network, where the megPAC approach delivered a similarly good match between MEG and fMRI networks. In contrast to the spatially more confined networks, these two networks encompass brain regions that are quite far apart. As a consequence, the principles of long‐range communication being mediated by low frequencies (Stein & Sarnthein, 2000) and of local communication by gamma oscillations (Buzsaki & Wang, 2012) are expected to play a more important role. This could explain why the megPAC approach, which considers both the local high gamma as well as the long‐range low‐frequency information, yielded a similar spatial correspondence for the DMN and right frontoparietal network.

This finding also indicates that there may be different electrophysiological underpinnings between the anatomically more focal RSN and those that are distributed across the cortex. For the local networks, communication mostly takes place within one frequency band via the amplitude of this frequency band. In contrast, for the distributed networks such as the DMN, communication is mediated via the principles of synchronized gating. In line with this finding on the DMN, it was recently shown that both amplitude and phase contain information relevant for understanding the resting brain (Siems & Siegel, 2020).

### 4.2 Frequency‐specificity of the Envelope‐ICA and Envelope‐SVD results

In the previous literature on Envelope‐ICA‐based RSN extraction from EEG or MEG, the activity of each RSN network was ascribed to a particular frequency band within each study—with findings not always converging across studies. For example, the DMN has been ascribed to both alpha and theta frequencies (Brookes et al., 2011; de Pasquale et al., 2010). When looking at the bootstrapped results in Figure 4, it was not the case that there was a single dominant frequency band for each RSN with the Envelope‐ICA approach. On the other hand, there is a clear frequency specificity for almost all networks and particularly for those which are anatomically and functionally confined with the Envelope‐SVD approach: The frontal network is best captured by alpha‐frequencies, the motor by theta, the visual by alpha and beta, and the parietal network by beta frequencies. The identified prominence of alpha and beta oscillations for the formation of RSNs is consistent with theory. Alpha oscillations are most prominent during rest and are linked with attentional processes (Klimesch, 2012). Beta oscillations on the other hand, signal the status‐quo (Engel & Fries, 2010). It is also broadly consistent with the previous literature on RSN extraction from EEG or MEG that have identified alpha and beta as the main frequency components (Brookes et al., 2011; de Pasquale et al., 2010; Hipp et al., 2012). At the same time, each study has ascribed the activity of each RSN network to a particular but often different frequency band. Our own experiments show that these non‐consistent previous findings on different dominant frequency components for a given RSN when applying the Envelope‐ICA can at least partially be explained by the variability in the ICA due to the dependence on the initial seed for optimization (Hyvarinen, 1999).

One further indication that the previously identified frequency specificity of the RSN might be a result of a bias was provided by Hipp and Siegel (2015), who investigated the frequency specificity of whole‐brain correlation of resting‐state MEG data. They identified a bias by the signal‐to‐noise ratio (SNR) of the different frequencies toward alpha and beta, which, if corrected, leads to an fMRI MEG correspondence over a wider range of frequencies. We also correct for the different SNR within frequencies by normalizing each frequency map independently by its cumulative density. Thus, our finding that the RSNs are not only best described by alpha and beta, but across the whole tested frequency range for the Envelope‐SVD approach aligns with the findings of Hipp and Siegel (2015).

When using ICA, it became apparent that for the MEG data the initial seed has a large influence, making the results not reproducible across different runs. Even using a stabilization procedure such as ICASSO (Himberg et al., 2004) did not improve the reproducibility across ICASSO runs—using previously proposed parameters for fMRI (Nugent et al., 2015). Our results indicate that different frequency components can be identified as the best match for a RSN with ICA just because (i) there was a different initial seed for the ICA and (ii) the RSNs obtained from different frequency bands do not differ too much. On the other hand, the ICA results for the fMRI are reproducible based on the current literature. To achieve this, a probabilistic ICA is implemented in Melodic, which has under the assumption of non‐Gaussian sources and Gaussian noise a unique solution (Beckmann & Smith, 2004). However, for the fMRI data not a temporal, but a spatial ICA is applied, so it is not clear if this approach is transferable to the MEG data. Based on the current literature on ICA, it is important to use an ICA approach optimized for the data. In line with this, Nugent et al. have proposed a multiband‐ICA approach, which estimates the ICA across all bands together. With this technique, it is possible to determine the shared loading across frequency bands—thus providing information on the frequency interaction (Nugent et al., 2017). Interestingly, they report that there is not one single frequency band best capturing the analyzed motor network.

In addition, one has to keep in mind that the current ICA approach enforces independence in the temporal domain but not in the frequency domain. Therefore, this approach may be ill‐suited to identify frequency‐specific RSNs. To obtain frequency‐specific information one should aim for independence in the frequency domain and factorize appropriate matrices to yield spectro‐spatial components (Hyvarinen et al., 2010). Future work should investigate this conjecture.

### 4.3 Choice of filter for Hilbert envelope

Before calculating the Hilbert transform for the Envelope‐ICA and Envelope‐SVD, the data have to be band‐pass filtered within the frequency bands of interest. When doing so the question arises whether the band‐pass is chosen in small enough frequency bands to correct for the natural 1/f power decrease in MEG data. Within our tests, using a wide‐band filter provided better correspondence to the fMRI networks. One potential explanation for this unexpected result could be that the wide band filtering emphasizes the lower frequencies, which also seem to be mainly driving the RSNs. On the other hand, the choice of an IIR or FIR filter did not seem to influence the results to a large degree.

### 4.4 Limitations

Within our study, we only included right‐handed young male subjects to avoid one additional source of variability within our data set. On the other hand, this means that our result might not transfer to all other populations. In addition, we did not control for head movement during the MEG recordings, because with the Elekta‐Neuromag system this is only possible when simultaneously using Maxfilter. However, as we have previously shown, maxfiltering can lead to unpredictable and uncontrollable changes in the frequency spectrum (Kandemir et al., 2020). Therefore, we refrained from continuous head tracking and only during the visual artifact rejection checked for movement artifacts. Ideally, this is something which future studies control for.

In general, source‐reconstructed MEG and EEG data are prone to source leakage (Palva & Palva, 2012), that is, nearby sources are correlated due to the imperfect source reconstruction. One common way to reduce source leakage is by orthogonalizing the envelope data (see, e.g., Hipp et al. (2012); Colclough et al. (2015)). We did not do this for the current paper, because it was also not applied in the original Brookes et al.'s (2011) paper and still the RSNs were well obtained with ICA. For the megPAC approach and the Envelope‐SVD approach, this problem is reduced by the application of the noise projectors, which are based on the same forward and inverse solution. Thereby the leakage inherent in the source reconstruction is also present in the noise data and thus reduced by this noise projector.

### 4.5 Comparison to previous studies

There have been several studies that investigated the test–retest reliability of different estimates of resting‐state connectivity (Colclough et al., 2016; Dimitriadis et al., 2018; Garces et al., 2016). These studies have provided important guidelines on the choice of connectivity measures. Overall, amplitude‐correlation based methods were more reliable than purely phase‐based methods. Compared to our study a direct comparison to the fMRI RSNs has been missing, that is, the aim of those older studies was not to identify networks as similar as possible to the fMRI RSNs but to provide guidelines on connectivity measures in general. This is also the reason why we had to limit our comparison to Envelope‐ICA/SVD and megPAC.

At the same time, the focus of our study was on data‐driven methods, as seed‐based approaches necessarily involve a human element that is impossible to control for. Additional data‐driven approaches involve finding time‐resolved networks (Baker et al., 2014; Cribben et al., 2012; Shappell et al., 2019; Vidaurre et al., 2018; Yaesoubi et al., 2015; Yaesoubi et al., 2018). The methods used to study time‐resolved activity vary widely in their underlying statistical assumptions as well as biological details (Lurie et al., 2020). Furthermore, source‐level network estimation using the time‐resolved methods employs a limited number of regions of interest based on atlases. Both the spatial reduction and statistical assumptions of the time‐resolved methods make them difficult to compare with ICA‐based fMRI networks—even though the HMM‐based approach provided very similar networks compared to the fMRI RSNs (Baker et al., 2014; Sitnikova et al., 2018; Vidaurre et al., 2016). However, the aim of our study was to identify markers of the static RSNs. A systematic evaluation of the time‐resolved approaches needs to be left for future research, but provided that MEG actually affords the high‐temporal resolution, these approaches make use of the distinct advantages of MEG. This also indicates a need to move away from trying to obtain the same RSNs as fMRI but focus more on features, which are uniquely identifiable with MEG. Still, the direct comparison between fMRI and MEG as done in the present study is a necessary step to make MEG accepted for the identification of RSN as known from the fMRI literature.

### 4.6 Recommendation for data‐driven RSN analysis

Based on our systematic comparison the recommendation for extracting data‐driven RSN from MEG data is to use the Envelope‐SVD approach, because it yielded significantly better correspondence for most fMRI‐RSN and in the two remaining cases, it was similar to the other approaches (i.e., no significant difference). As this approach requires the extraction of the Hilbert‐Envelope in different frequency bands, the filter setting is also a relevant choice and here the recommendation is to use a wide‐band, that is, a filter that encompasses the whole frequency band of interest, IIR filter.

## 5 CONCLUSION

In summary, the novel Envelope‐SVD approach successfully extracted the RSN from electrophysiological recordings. Consistent with theory, this approach identified a dominant frequency component for the RSNs. For five RSNs, in particular those, which are anatomically confined, the Envelope‐SVD yielded significantly higher correspondence to fMRI‐RSNs compared to other data‐driven methods. The exceptions were the DMN and the right frontoparietal network, where megPAC yielded a similar correspondence by incorporating the principles of long‐ and short‐range communication. Overall, these findings enhance our understanding on the electrophysiological underpinnings of the fMRI‐RSN.

## FUNDING INFORMATION

Funded by the Volkswagen Foundation (Lichtenberg program 89387).

## CONFLICT OF INTEREST STATEMENT

The authors declare no conflicts of interest.

## Supporting information


**FIGURE S1:** **Highest correspondence for three resting‐state networks obtained from MEG and fMRI recordings using the three different approaches**. For each MEG method, the RSN with the largest spatial correspondence to the fMRI‐RSN across all frequency bands and extracted networks is displayed. The row represents the respective RSN, while the columns show the spatial extent comparing fMRI to each of the three MEG approaches. The relevant frequencies are for the Envelope‐SVD and Envelope‐ICA approach and are written on the left of each network. The networks are all thresholded at 0.85 based on the probability distribution of the network's values.
Figure S2: **Highest correspondence for four resting‐state networks in each frequency band for the Envelope‐SVD approach of one run**. The box indicates the optimal frequency for each network. Legend see Figure S1.
Figure S3: **Highest correspondence for four resting‐state networks in each frequency band for the Envelope‐ICA approach for one run**. The box indicates the optimal frequency for each network. Legend see Figure S1.

## Data Availability

The MATLAB codes are available on https://github.com/FlorinNeuro/. (https://www.bihealth.org/en/translation/network/digitalmedicine/bihcharite‐virtual‐research‐environment). The raw data can be found at the following location: https://search.kg.ebrains.eu/?category=Dataset.
