# Supplementary material for: Data‐driven MEG analysis to extract fMRI resting‐state networks
Source: Hum Brain Mapp. 2024 Mar 6;45(4):e26644. doi: 10.1002/hbm.26644 (PMC10915736; doi:10.1002/hbm.26644)

**Supplementary Material**

**Figure SI 1: Highest correspondence for three resting state networks obtained from MEG and fMRI recordings using the three different approaches**

For each MEG method, the RSN with the largest spatial correspondence to the fMRI-RSN across all frequency bands and extracted networks is displayed. The row represents the respective RSN, while the columns show the spatial extent comparing fMRI to each of the three MEG approaches. The relevant frequencies are for the Envelope-SVD and Envelope-ICA approach are written on the left of each network. The networks are all thresholded at 0.85 based on the probability distribution of the network’s values.


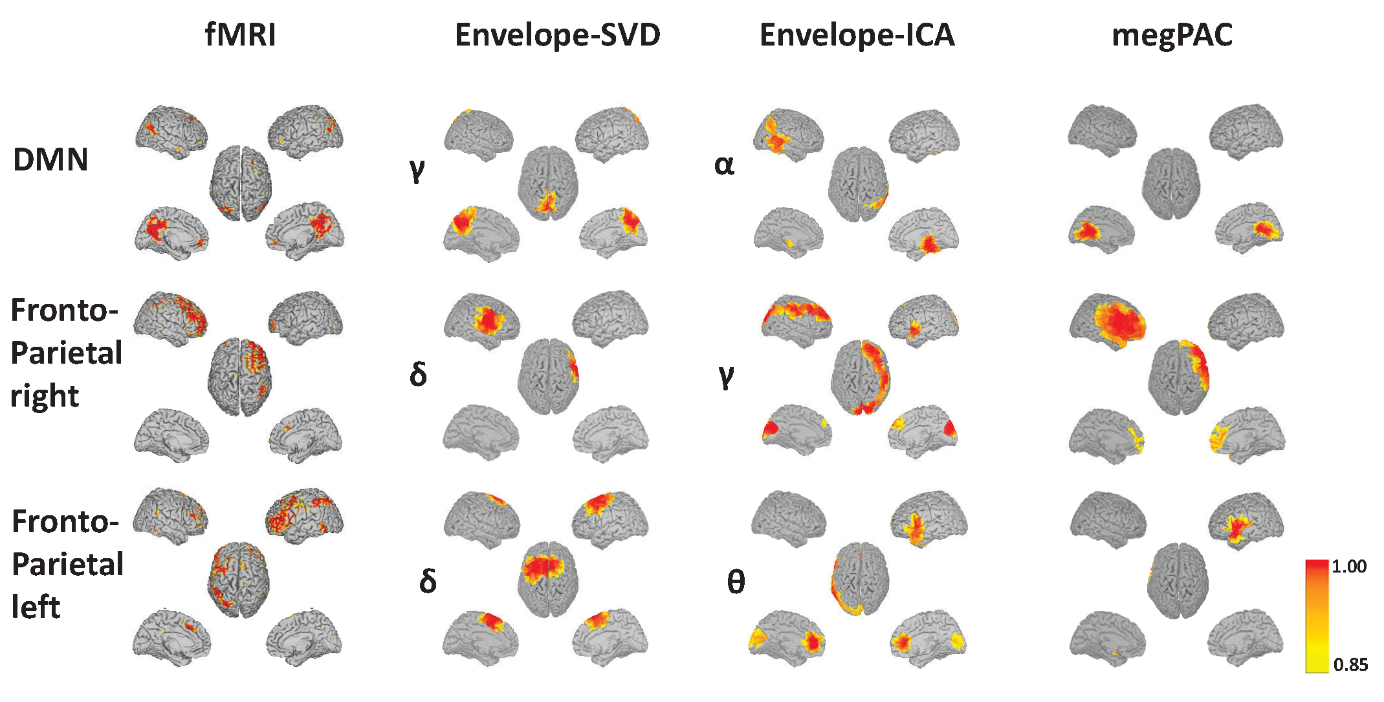


**Figure SI 2: Highest correspondence for four resting state networks in each frequency band for the Envelope-SVD approach of one run**

The box indicates the optimal frequency for each network. Legend see Figure SI 1.


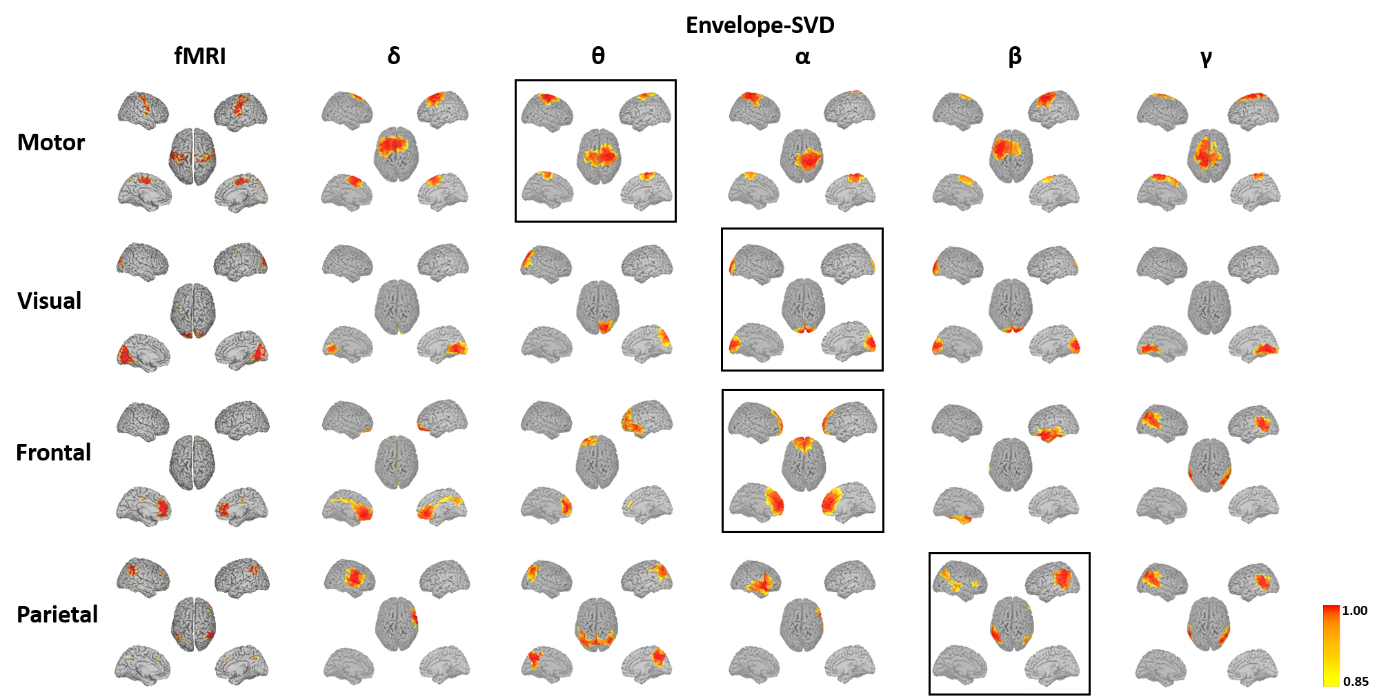


**Figure SI 3: Highest correspondence for four resting state networks in each frequency band for the Envelope-ICA approach for one run**

The box indicates the optimal frequency for each network. Legend see Figure SI 1.


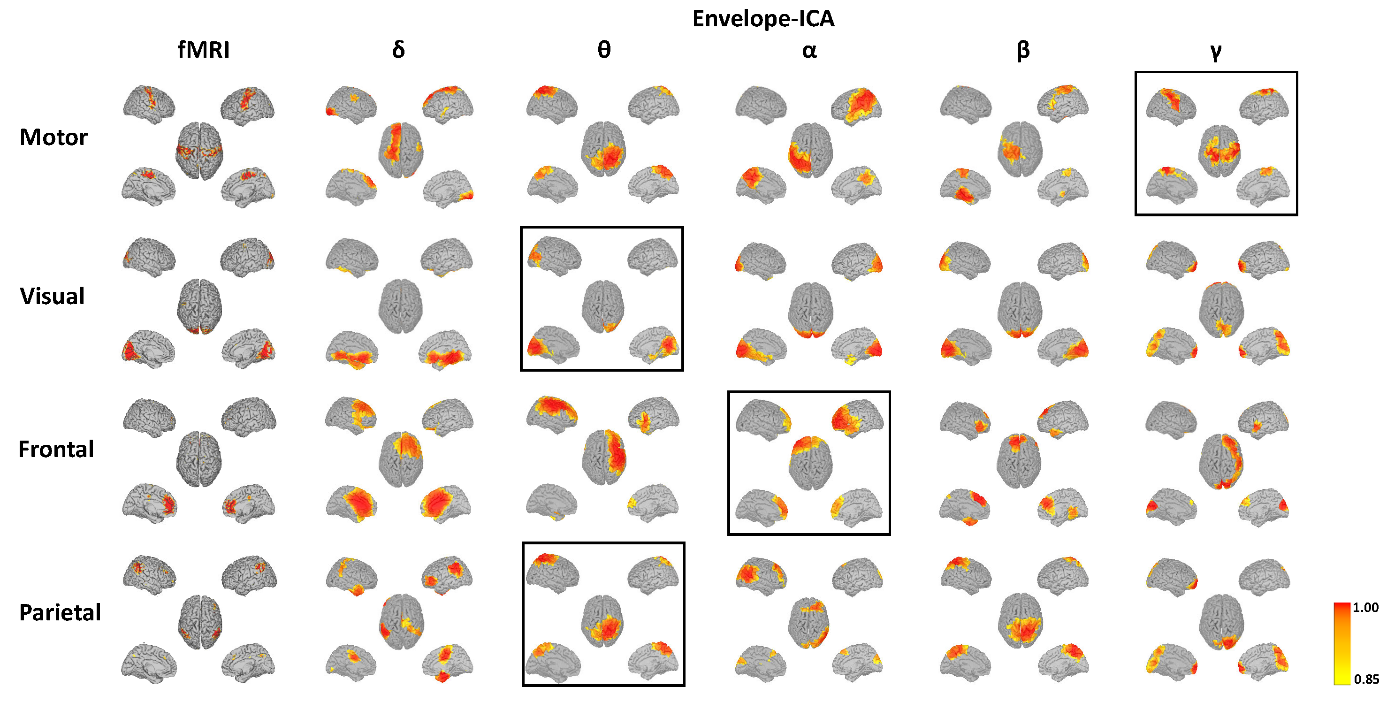

Supplement: Supplementary file 1 — FIGURE S1: Highest correspondence for three resting‐state networks obtained from MEG and fMRI recordings using the three different approaches. For each MEG method, the RSN with the largest spatial correspondence to the fMRI‐RSN across all frequency bands and extracted networks is displayed. The row represents the respective RSN, while the columns show the spatial extent comparing fMRI to each of the three MEG approaches. The relevant frequencies are for the Envelope‐SVD and Envelope‐ICA approach and are written on the left of each network. The networks are all thresholded at 0.85 based on the probability distribution of the network's values. Figure S2: Highest correspondence for four resting‐state networks in each frequency band for the Envelope‐SVD approach of one run. The box indicates the optimal frequency for each network. Legend see Figure S1. Figure S3: Highest correspondence for four resting‐state networks in each frequency band for the Envelope‐ICA approach for one run. The box indicates the optimal frequency for each network. Legend see Figure S1. [file HBM-45-e26644-s001.docx]
